# Parental Legacy in Insects: Variation of Transgenerational Immune Priming during Offspring Development

**DOI:** 10.1371/journal.pone.0063392

**Published:** 2013-05-20

**Authors:** Ute Trauer, Monika Hilker

**Affiliations:** Institute of Biology – Applied Zoology/Animal Ecology, Freie Universität Berlin, Berlin, Germany; Swedish University of Agricultural Sciences, Sweden

## Abstract

In insects, a parental immune challenge can prepare and enhance offspring immune activity. Previous studies of such transgenerational immune priming (TGIP) mainly focused on a single offspring life stage. However, different developmental stages may be exposed to different risks and show different susceptibility to parental immune priming. Here we addressed the question (i) whether TGIP effects on the immunity of *Manduca sexta* offspring vary among the different developmental offspring stages. We differentiated between unchallenged and immunochallenged offspring; for the latter type of offspring, we further investigated (ii) whether TGIP has an impact on the time that enhanced immune levels persist after offspring immune challenge. Finally, we determined (iii) whether TGIP effects on offspring performance depend on the offspring stage. Our results show that TGIP effects on phenoloxidase (PO) activity, but not on antibacterial activity, vary among unchallenged offspring stages. In contrast, TGIP effects on PO and antibacterial activity did not vary among immunochallenged offspring stages. The persistence of enhanced immune levels in immunochallenged offspring was dependent on the parental immune state. Antibacterial (but not PO) activity in offspring of immunochallenged parents decreased over five days after pupal immune challenge, whereas no significant change over time was detectable in offspring of control parents. Finally, TGIP effects on the developmental time of unchallenged offspring varied among stages; young larvae of immunochallenged parents developed faster and gained more weight than larvae of control parents. However, offspring females of immunochallenged parents laid fewer eggs than females derived from control parents. These findings suggest that the benefits which the offspring gains from TGIP during juvenile development are paid by the adults with reduced reproductive power. Our study shows that TGIP effects vary among offspring stages and depend on the type of immunity (PO or antibacterial activity) as well as the time past offspring immune challenge.

## Introduction

Insects defend themselves against pathogens and parasitoids by their innate immune system. The effectiveness of this innate immunity is shaped by several factors ranging from abiotic parameters [1] to the type of food ingested ([2]–[4] and references therein) or the immune challenges and risks experienced before [5].

Numerous studies show that the experience of a microbial infection or parasitic attack can improve the insects immune response to a subsequent exposure to pathogens or parasitoids [6]–[8]. Such “immune priming” by a first immune challenge may be beneficial at environmental conditions at which subsequent encounters of pathogens or parasitoids are likely and have an impact on the fitness of the primed organism [9]. A priming effect of an immune challenge on later immune responses can be traced within a generation from an early juvenile stage to an elder one [6], [10], from a larval stage to the adult [7], [11], [12], and also within the adult stage [13]. Several studies showed that immune priming may even persist in the offspring generation. When pathogenic threats experienced by the parental generation remain until the offspring generation, transgenerational immune priming (TGIP) may improve offspring survival. Some TGIP studies demonstrated that an immune challenge of parental insects in their larval stage can prime immune responses of a larval offspring stage [14]–[18], while other studies showed that immunochallenged adult parental insects can significantly prime the immune defence of their offspring in the adult stage [19]–[22].

The studies of TGIP mentioned above focused on the analyses of a priming effect on the immune state of a single, particular life stage rather than tracking the priming effect across different stages of the offspring generation from larvae to adults. However, in unchallenged insects, the different ontogenetic life stages are known to show different immune states [23]. Since the various life stages are often exposed to different risks, TGIP might differentially affect the immune state of different offspring stages and thus, be of varying relevance throughout the life of an organism.

Even though an insect may benefit from immune priming by improved resistance against pathogens or parasitoids, maintenance and use of immune functions are well known to be costly (reviewed in [24]). Costs and benefits of TGIP may differ according to the risks experienced by the different developmental stages and the energy needed to establish and maintain a primed immune state. For example, young larvae that usually suffer high risks of being parasitized or preyed upon may be affected differently by TGIP than elder larvae. Furthermore, pupae that are living in the soil and are exposed to another microbial environment than stages living above ground may be influenced differently by TGIP than larvae and adults. However, so far TGIP effects have not been traced yet from hatching offspring larvae to offspring adults. A few recent studies have shown that TGIP is paid with worse offspring performance expressed in terms of e.g. enhanced developmental times until adult eclosion, reduced offspring weight, reduced F1 fecundity, and even enhanced offspring mortality [16], [21], [22].

In this study we used the tobacco hornworm *Manduca sexta* as a model system in order to elucidate TGIP effects on immunity and performance of the offspring throughout its entire development from the larval stage to the adult. Development of the larvae which feed upon the leaves of mainly solanaceous host plants takes five instars; then the prepupal wandering stage digs a few centimetres into the soil for pupation, and after about 21 days (depending on abiotic conditions) the adult moths eclose from the soil [25]. The 2^nd^ and 3^rd^ instar larvae suffer a high mortality (approx. 90%) in the field due to parasitism and predation [26]–[28]. Hence, survival to pupation is strongly influenced by the risk that young larvae experience by natural enemies [27], [29]. The immune system of *M. sexta* larvae is known to be able to respond to priming by a previous immune challenge. An injection of a non-pathogenic bacterium into the 5^th^ instar of *M. sexta* larvae caused stronger immune responses in the same instar and better survival of a subsequent pathogen infection [10]. However, transgenerational immune priming effects have not been studied yet in *M. sexta*.

We here addressed the following questions: (i) Does TGIP affect the immunity of the various *M. sexta* offspring stages (larval instars, pupae, adults) differently? We studied this question by differentiating between unchallenged and challenged immune states of the offspring. (ii) Does TGIP affect the persistence of enhanced immune activity levels after offspring immune challenge? Are enhanced immune activity levels of immunochallenged primed offspring individuals maintained for a longer time than immunity levels of immunochallenged non-primed ones? (iii) Does TGIP affect performance of the various offspring developmental stages differently?

In order to investigate TGIP effects on the different offspring stages, the parental generation was challenged in its pupal stage by injection of peptidoglycan (PGN), a non-pathogenic surface molecule of bacteria that was dissolved in phosphate buffered saline (PBS). For control, we analysed the immune state of the offspring of PBS-control injected parents and of untreated (naive) parental individuals. When studying immunity of immunochallenged offspring of these three parental groups, the offspring individuals were subjected to the same three different types of treatments as the parental generation. *M. sexta* immune defence levels were determined by measuring phenoloxidase (PO) activity and antibacterial activity in haemolymph samples.

Binding of bacterial PGN to insect pattern-recognition proteins leads to activation of different immune responses such as increase of PO activity and antibacterial activity [30]–[33]. The activation of the PO cascade and induction of the synthesis of antimicrobial peptides (AMPs) have intensively been investigated in *M. sexta* (reviewed in [34]–[36]). PO is an important enzyme that is involved in melanisation and encapsulation of pathogenic or parasitic invader [31], [37]. Furthermore, oxidation reactions catalysed by PO lead to the formation of toxic compounds that may contribute to the killing of invading pathogens [38], [39]. Induction of the PO cascade follows a complex temporal pattern depending on the type of immune challenge; PO activity can be upregulated within 1 h after a pathogen attack and is then maintained for more than 24 h [40], [41]. PO activation and melanisation in response to microbial exposure can occur faster than AMP synthesis [36]. The synthesis of specific AMPs is induced in the insect haemolymph 2–48 h after an immune challenge, and an induced level of antimicrobial peptides can be maintained for 14 days [40], [41].

Our findings show that TGIP effects strongly depend on the offspring developmental stage studied, on the immune parameters that were analysed (PO activity, antibacterial activity), on the offspring immune state (unchallenged or challenged immune state), and the time past immune challenge of the offspring.

## Results

### TGIP Effects on Immunity of Unchallenged Offspring

**Figure 1:**
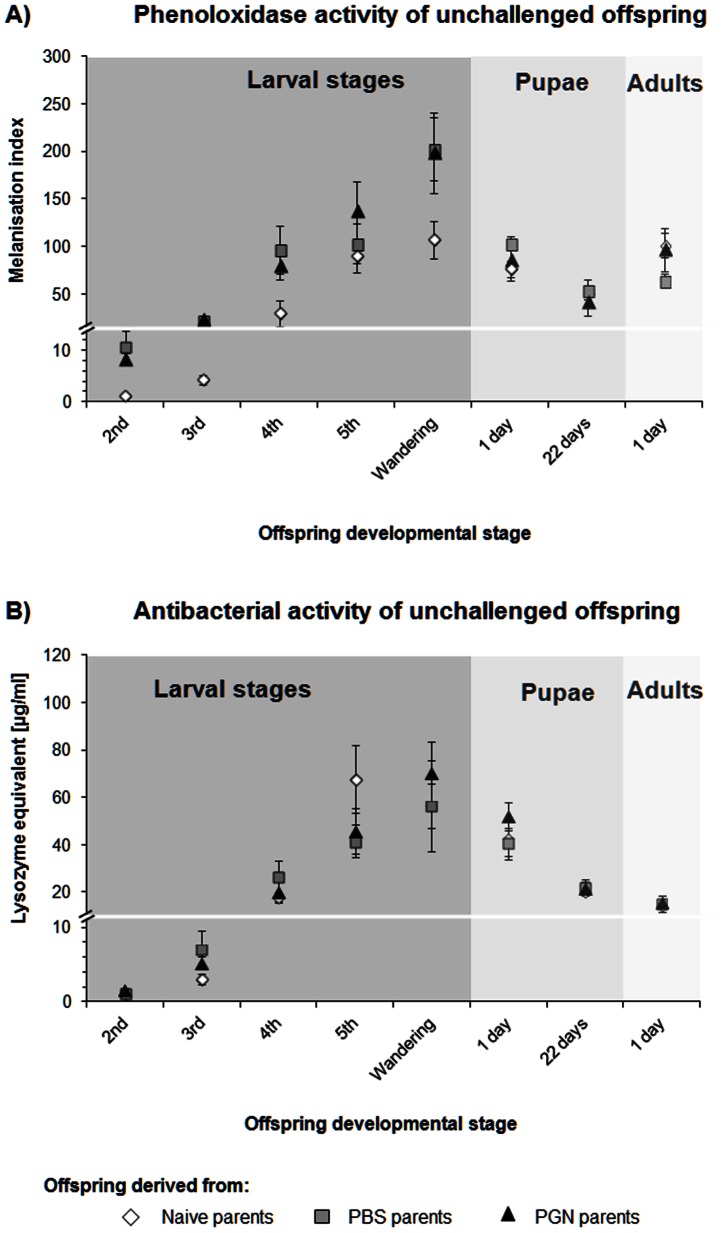
Transgenerational immune priming effects on immune activity of unchallenged *Manduca sexta* offspring. **A**) Phenoloxidase activity and **B**) antibacterial activity (lysozyme activity equivalent, *Micrococcus luteus*) were measured during offspring development at the first day of each developmental stage and in 22-day-old pupae. Female and male parents received a priming treatment in their pupal stage: Naive) untreated, PBS) control-injected with phosphate buffered saline, PGN) injected with peptidoglycan. If the symbol for offspring of naive parents is not visible, it is overlaid by another symbol. Means ± SE are given. *N* = 9 samples of each developmental stage from each parental group. Differences between the parental priming treatments and the offspring developmental stages were compared by 2-way-ANOVA (Table 1) and post-hoc analysis Tukey tests (Table S3 for PO activity, table S4 for antibacterial activity in File S1).

In order to investigate whether the impact of the parental immune state on the immunity of unchallenged offspring varies with the developmental stage of the offspring, we measured PO and antibacterial activity of offspring individuals in their larval, pupal and adult stages in dependence of the immune treatment of their parents (three parental groups: PGN injected, PBS-control injected, naive parents) (Fig. 1) (immunity levels of parental treatment groups: Table S1, S2 in File S1).

PO activity of the unchallenged offspring generation was significantly affected by the parental treatment (Table 1, two-way-ANOVA, factor parental treatment, *P*<0.001) and the offspring stage (Table 1, two-way-ANOVA, factor offspring stage, *P*<0.001). The impact of the parental immune state on the immunity of unchallenged offspring was dependent on the developmental stage of the offspring (Table 1; two-way ANOVA, significant interaction between parental treatment × offspring stage, *P*<0.001). PO activity of the unchallenged offspring of all three parental groups was lowest in the 2^nd^ instar larvae, reached its peak in the larval wandering stage, decreased during the pupal stage and increased again in the adult stage (Fig. 1A). Unchallenged larvae of the 2^nd^ to 4^th^ instar that were derived from parents treated with a PGN injection or PBS-control injection showed a significantly higher PO activity than unchallenged larvae of naive parents (Table S3, post-hoc Tukey test in File S1). PO activity of offspring larvae of PBS-control injected or PGN-immunochallenged parents was always about as strong as the PO activity in the following larval stage of offspring individuals derived from unchallenged parents (Fig. 1A, Table S3 in File S1). No such effects of the parental treatment on offspring PO activitiy were detectable in the other offspring developmental stages studied.

**Table 1:** Unchallenged offspring immunity.

Source	PO activity	post hocTukey test	Antibacterialactivity	post hocTukey test
Parental treatment	df = 2		df = 2	
	MS = 1.257		MS = 0.008	
	F = 16.040		F = 108.434	
	***P*** **<0.001**	Naive – PBS: ***P*** **<0.001**	P = 0.267	
		Naive – PGN: ***P*** **<0.001**		
		PBS – PGN: *P* = 0.992		
Offspring stage	df = 7		df = 7	
	MS = 4.351		MS = 0.679	
	F = 55.533		F = 1.328	
	***P*** **<0.001**		***P*** **<0.001**	Table S4 in File S1
Parental treatment x	df = 14		df = 14	
Offspring stage	MS = 0.286		MS = 0.005	
	F = 3.655		F = 0.752	
	***P*** **<0.001**	Table S3 in File S1	*P* = 0.720	

Statistical evaluation (two-way ANOVA) of the priming effects on phenoloxidase (PO) and antibacterial (AMP) activity (lysozyme activity equivalent, *Micrococcus luteus*) of unchallenged *Manduca sexta* offspring from differently treated parents (naive, PBS, PGN) (compare Fig. 1 and Tables S3, S4 for post hoc test data in File S1).

Data were Box-Cox transformed prior to analysis in order to reach normal distribution PO = PO^∧^0.185, AMP = AMP^∧^0.095. Significant *P*-levels are shown in bold.

Antibacterial activity of unchallenged offspring increased during larval development and decreased thereafter in the pupal and adult stage (Fig. 1B). This general trend was found in the offspring of all three parental groups. Hence, antibacterial activity was dependent on the developmental stage (Table 1, two-way ANOVA, factor offspring stage, *P*<0.001, Table S4, post-hoc Tukey test in File S1). However, antibacterial activity of all developmental stages showed no differences in dependence of the parental treatments (Table 1, two-way ANOVA, factor parental treatment, *P* = 0.267).

Hence, while TGIP effects on PO activity of unchallenged offspring varied with the developmental offspring stage studied, TGIP effects on antibacterial activity did not.

### TGIP Effects on Immunity of Challenged Offspring

**Figure 2:**
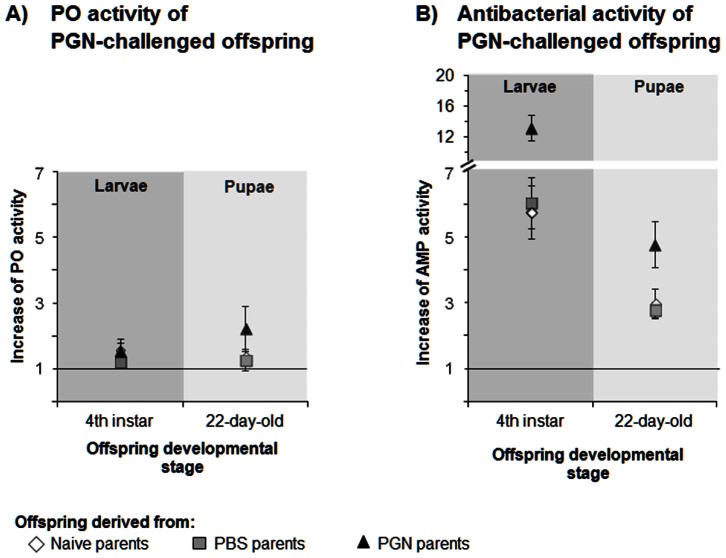
Transgenerational immune priming effects on the increase of immune activity of *Manduca sexta* offspring larvae and pupae one day after offspring immune challenge by PGN. A) Increase of phenoloxidase (PO) activity and B) increase of antibacterial (AMP) activity (lysozyme activity equivalent, *Micrococcus luteus*) were measured in 4^th^ instar larvae and 22-day-old pupae one day after offspring immune treatment. Female and male parents received a priming treatment in their pupal stage: Naive) untreated, PBS) control-injected with phosphate buffered saline, PGN) injected with peptidoglycan. If the symbol for offspring of naive parents is not visible, it is overlaid by another symbol. Increase of immune activity was measured as increase = (Activity after PGN treatment of the offspring)/(Mean activity of unchallenged offspring); value 1 is labelled by a line that indicates no change in immune activity after offspring challenge. Please note the comparable scales for increases which show the immunity and visualise the strong priming effects on offspring AMP activity, but the lack of effects on PO activity in the offspring. Mean values ± SE are given. *N = *9 individuals of each developmental stage from each parental group. Means of absolute data of PGN- and PBS-treated offspring are shown in Table S5 in File S1. Differences between the parental priming treatments and the offspring developmental stages were compared by 2-way-ANOVA (Table 2). Statistical evaluation of priming effects on the increase of immunity after offspring immune challenge by PBS is shown in Table S6 in File S1.

In order to investigate whether the impact of the parental immune state on the immunity of PGN-challenged offspring varies with the developmental stage of the offspring, we measured PO and antibacterial activity of offspring individuals after an immune challenge. The offspring individuals of the three parental groups were challenged with a PGN injection in their 4^th^ larval stage. Furthermore, other offspring individuals of the three parental groups were challenged in the same way in the pupal stage (21-day-old pupae). We measured offspring PO and antibacterial activity one day after treatment (L4-larvae, 22-day-old pupae) and determined how these immune parameters differ from immunity of the respective unchallenged stages. Figure 2 shows how the PO or antibacterial activity level changed after an immune challenge when compared to the PO or antibacterial activity level of unchallenged offspring individuals (value 1 =  no change).

An immune challenge of offspring larvae and pupae by PGN-injection did not lead to an increase of PO activity as compared to the activity in unchallenged larval offspring individuals (Fig. 2A, values about 1 to 2). This finding was independent of the parental group the offspring was derived from (Table 2, two-way ANOVA, factor parental treatment, *P* = 0.469, Table S5, absolute data in File S1).

**Table 2:** PGN-challenged offspring immunity.

Source	PO activity	Antibacterialactivity	post hoc Tukey test
Parental treatment	df = 2	df = 2	
	MS = 0.053	MS = 0.006	
	F = 0.768	F = 16.555	
	*P* = 0.469	***P*** **<0.001**	Naive - PBS: *P* = 0.959
			Naive - PGN: ***P*** **<0.001**
			PBS - PGN: ***P*** **<0.001**
Offspring stage	df = 1	df = 1	
(L4, pupae)	MS = 0.0007	MS = 0.021	
	F = 0.010	F = 55.761	
	*P* = 0.922	***P*** **<0.001**	
Parental treatment x	df = 2	df = 2	
Offspring stage	MS = 0.004	MS = 0.0006	
	F = 0.062	F = 1.465	
	*P* = 0.940	*P* = 0.241	

PGN = peptidoglycan. Statistical evaluation (two-way-ANOVA) of priming effects on the increase of phenoloxidase (PO) and antibacterial (AMP) activity (lysozyme activity equivalent, *Micrococcus luteus*) of *Manduca sexta* offspring from differently treated parents (naive, PBS, PGN) one day after offspring PGN treatment in 4^th^ instar larvae and 22-day-old pupae (Fig. 2). Means ± SE of PO activities and antibacterial activities of PGN-challenged offspring individuals are shown in Table S5 in File S1. Statistical evaluation of priming effects on the increase of immunity after offspring immune challenge by PBS is shown in Table S6 in File S1.

Data were Box-Cox transformed prior to analysis in order to reach normal distribution PO = PÔ0.318, AMP = AMPˆ0.046. Significant *P*-levels are shown in bold.

For control, we also measured the PO activity of larvae and pupae that were PBS-control injected. No increase of PO activity was found in these larvae and pupae when compared to untreated ones of each parental treatment group (Table S5 in File S1). Their PO activity was neither affected by the parental treatment nor by the offspring stage (Table S6, two-way ANOVA in File S1).

In contrast, antibacterial activity of the offspring larvae and pupae was strongly induced one day after offspring PGN treatment (Fig. 2B). The intensity of induction of antibacterial activity in these offspring stages was dependent on the parental treatment (Table 2, two-way ANOVA, factor parental treatment, *P*<0.001). In larvae of control parents, the offspring PGN treatment led to an about 6-fold increase of antibacterial activity, whereas in larvae of PGN parents the offspring PGN treatment led to an about 13-fold increase of antibacterial activity (Fig. 2B, Table S5, absolute data in File S1). In 4^th^ instar larvae, the antibacterial activity after PGN treatment was stronger than antibacterial activity in all unchallenged offspring developmental stages, independent of the parental treatment (Table S5 in File S1). PGN-treated offspring pupae of control parents showed an about 3-fold increase of antibacterial activity, whereas PGN-treated offspring pupae of PGN-treated parents showed an about 5-fold increase of antibacterial activity; their antimicrobial activity was higher than in all unchallenged developmental stages (Fig. 2B, Table S5 in File S1). Hence, the parental PGN treatment significantly affected the level of induction of antibacterial activity in larvae and pupae (Table 2, two-way ANOVA, factor parental treatment, *P*<0.001). The immune levels significantly differed with the offspring stage studied (Table 2, two-way ANOVA, factor offspring stage, *P*<0.001). However, no significant interaction was detected between the parental treatment and offspring stage (Table 2, two-way ANOVA, factor parental treatment × offspring stage, *P* = 0.241).

For control, we also tested the antibacterial activity of larvae and pupae that were PBS-control injected. However, these larvae and pupae neither showed an increase of antibacterial activity (Table S5, absolute data in File S1) nor effects of parental treatment or offspring stage on the PBS-induced antibacterial activity of the offspring (Table S6, two-way ANOVA in File S1).

Hence, no indication was found that TGIP effects on immunochallenged offspring immunity (PO or antibacterial activity) vary with the ontogenetic offspring stage studied here.

### TGIP Effects on Persistence of Challenged Offspring Immunity

**Figure 3:**
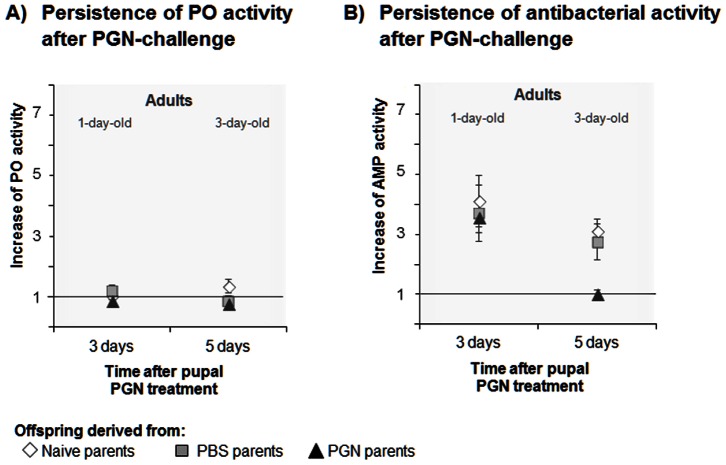
Persistence of transgenerational immune priming effects on the increase of immune activity of *Manduca sexta* offspring after offspring immune challenge by PGN in 21-day-old pupae. A) Increase of phenoloxidase (PO) activity and B) increase of antibacterial (AMP) activity (lysozyme activity equivalent, *Micrococcus luteus*) were measured in 1-day-old and 3-day-old adults, i.e. three and five days, respectively, after offspring immune treatment in 21-day-old pupae. Female and male parents received a priming treatment in their pupal stage: Naive) untreated, PBS) control-injected with phosphate buffered saline, PGN) injected with peptidoglycan. If the symbol for offspring of naive parents is not visible, it is overlaid by another symbol. Increase of immune activity was measured as increase = (Activity after PGN treatment of the offspring)/(Mean activity of unchallenged offspring); value 1 is labelled by a line that indicates no change in immune activity after offspring challenge. Please note the comparable scales for increases which show the immunity and visualise the strong priming effects on offspring AMP activity, but the lack of effects on PO activity in the offspring. Mean values ± SE are given. *N = *9 individuals of each developmental stage from each parental group. Means of absolute data of PGN- and PBS-treated offspring are shown in Table S5 in File S1. Differences between the parental priming treatments and the time intervals after offspring PGN treatment were compared by two-way ANOVA (Table 3) and post hoc analysis Tukey tests (Table S7 in File S1). Statistical evaluation of priming effects on the persistence of immunity after offspring immune challenge by PBS is shown in Table S8 in File S1.

We investigated how the parental immune challenge affects the persistence of enhanced immune activity in offspring that had been challenged by PGN. We analysed (i) for how long enhanced immune activity levels of offspring individuals are maintained after an immune challenge and (ii) how this depends on the parental immune challenge. We examined whether an increase of immune activity of offspring pupae after PGN challenge two days prior to adult emergence is still maintained in the offspring adults (Fig. 3). We measured immune activity levels of one set of adults that were 1 day old (i.e. 3 days after they have received a PGN injection in their pupal stage) and of another set of adults that were 3 days old (i.e. 5 days after they have received a PGN injection in their pupal stage). Figure 3 shows how the immune parameters of these adults differ from immunity of the respective unchallenged adults (value 1: no change).

As was found for PGN-challenged L4-larvae and the 22-day-old offspring pupae (Fig. 2), the PO activity of PGN-challenged 1- and 3-day-old offspring adults was not affected by parental treatment (Fig. 3A, Table 3, *P* = 0.102, Table S5 in File S1). However, antibacterial activity of the offspring adults was significantly affected by the parental treatment (Fig. 3B, Table 3, factor parental treatment, *P*<0.05, Table S5, absolute data in File S1). The increase of PGN-induced antibacterial activity in the offspring adults was dependent on the time after their immune challenge (Table 3, factor time after challenge, *P*<0.001). Five days after offspring immune challenge, antibacterial immune activity of offspring adults with PGN-challenged parents was the lowest (value = 1, i.e. no increase of immune activity) when compared to an about 3-fold increase of antibacterial activity in offspring adults with PBS-treated and control parents (Table S7, post hoc Tukey test in File S1).

**Table 3:** PGN-challenged offspring immunity.

Source	PO activity	Antibacterialactivity	post hoc Tukey test
Parental treatment	df = 2	df = 2	
	MS = 0.035	MS = 0.029	
	F = 2.399	F = 4.965	
	*P* = 0.102	***P*** **<0.05**	
Time after offspring	df = 1	df = 1	
challenge in pupal stage	MS = 0.003	MS = 0.077	
(3 d, 5 d)	F = 0.189	F = 12.938	
	*P* = 0.666	***P*** **<0.001**	
Parental treatment x	df = 2	df = 2	
Time after challenge	MS = 0.023	MS = 0.027	
	F = 1.570	F = 4.628	
	*P* = 0.219	***P*** **<0.05**	Table S7 in File S1

PGN = peptidoglycan. Statistical evaluation (two-way-ANOVA) of priming effects on the persistence of the increase of phenoloxidase (PO) and antibacterial (AMP) activity (lysozyme activity equivalent, *Micrococcus luteus*) three and five days after offspring pupal PGN treatment in 3-day-old and 5-day-old adults (Fig. 3). Means ± SE of PO activities and antibacterial (AMP) activities of PGN-challenged offspring individuals are shown in Table S5 in File S1. Statistical evaluation of priming effects on the persistence of enhanced immune activity after offspring immune challenge by PBS is shown in Table S8 in File S1. Compare Table S7 for post hoc test data in File S1.

Data were Box-Cox transformed prior to analysis in order to reach normal distribution PO = PÔ0.242, AMP = AMPˆ0.121. Significant *P*-levels are shown in bold.

Hence, the effect of the parental treatment on the persistence of antibacterial activity significantly differed with the time past offspring PGN-challenge (Table 3, factor parental treatment × time after challenge, *P*<0.05).

For control, we also tested PO and antibacterial activity of adults that were PBS-control injected in their pupal stage. Their immune parameters were independent of the parental treatment (Table S8, two-way ANOVA in File S1). The time after offspring PBS-challenge in the pupal stage had a significant effect on the PO-level of the resulting adults, but did not affect the antibacterial activity of the adults (Table S8 in File S1). Furthermore, no significant interactive effect of parental treatment × time past challenge on PO activity and antibacterial activity was found in adults that were challenged by PBS in the pupal stage (Table S8 in File S1). Thus, in contrast to the TGIP persistence effects on antibacterial activity observed after offspring PGN-challenge (Table 3, factor parental treatment × time after challenge, *P*<0.05), no such effects were detected for PBS-challenged offspring; the effect of the parental treatment on the persistence of PBS-induced antibacterial activity in the offspring did not depend on the time past offspring PBS challenge (Table S8 in File S1).

### TGIP Effects on Weight and Developmental Time of Unchallenged Offspring

**Figure 4:**
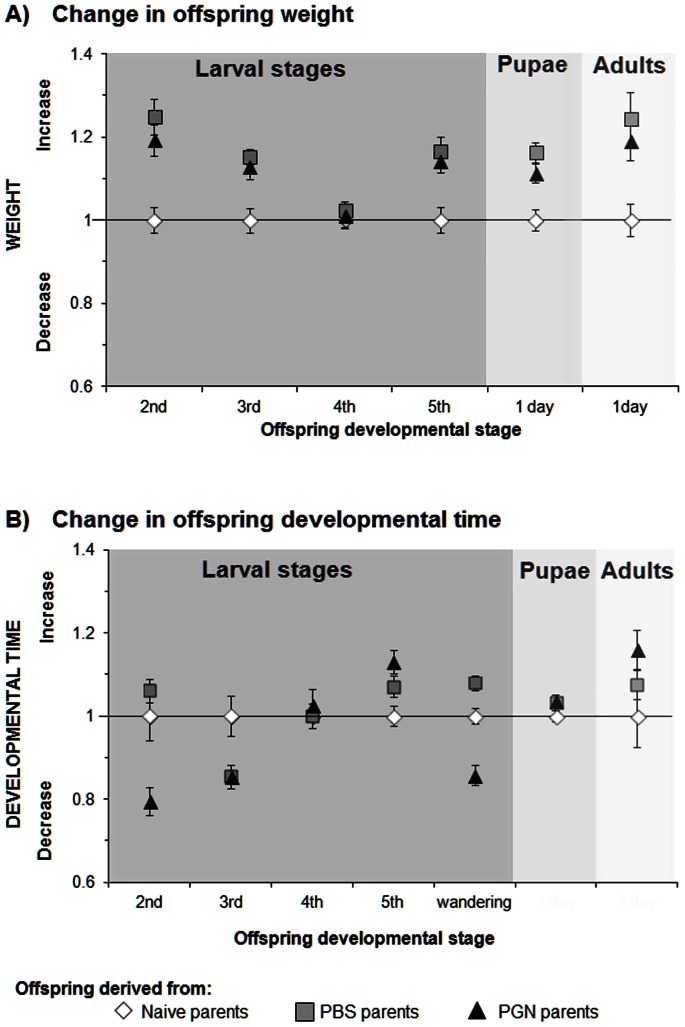
Transgenerational immune priming effects on weight and developmental time of unchallenged *Manduca sexta* offspring. Change (increase/decrease) in weight (**A**) and developmental time (**B**) of offspring derived from naive parents and offspring of PBS- or PGN-treated parents were calculated for each offspring stage of each parental group as ratio = (Individual weight or developmental time of offspring of the respective parental treatment group)/(Mean weight or developmental time of offspring derived from naive parents). Larval weight was determined at the last day of each instar, whereas pupal and adult weight was determined at the first day of the respective stage. Value 1 is labelled by a line that indicates no change in weight or developmental time compared to offspring derived from naive parents. Female and male parents received a priming treatment in their pupal stage: Naive) untreated, PBS) control-injected with phosphate buffered saline, PGN) injected with peptidoglycan. If the symbol for offspring of naive parents is not visible, it is overlaid by another symbol. Mean ratios ± SE are given. *N = *18 individuals of each developmental stage except for adults *N* = 9 from each parental group. Means of absolute data of offspring weight and developmental time are shown in Table S9 in File S1. Differences between the parental priming treatments and the offspring developmental stages were compared by Generalized linear model (Table 4) and post hoc analyses *U*-test (Table S10 weight, table S11 developmental time in File S1).

In order to elucidate the impact of a parental priming treatment on the performance of the different offspring stages, we compared developmental times and weight of juvenile and adult stages of unchallenged offspring of the three parental treatment groups. Figure 4 shows how weight and development times of the different offspring stages of PBS- or PGN-treated parents differed from the performance parameters of the respective stages derived from unchallenged parents (value 1 =  no difference).

The parental treatment significantly affected weight of the offspring stages. Except for 4^th^ instar larvae, offspring of PBS- or PGN-injected parents gained more weight than offspring of naive parents (Fig. 4A, Table 4, factor parental treatment, *P*<0.001, Table S9, absolute data in File S1). The changes in weight after PBS and PGN treatment were dependent on the offspring stage studied (Table 4, factor offspring stage, *P*<0.001, Table S10, post-hoc Tukey test in File S1). However, no significant interaction was detected between the parental treatment and offspring stage (Table 4, factor parental treatment × offspring stage, *P*<0.143). Therefore, the effects of the parental immune treatment on offspring weight did not significantly change with the ontogenetic offspring stages studied.

**Table 4:** Performance of unchallenged offspring.

Source	Weight	post hoc *U*- test	Developmental Time	post hoc *U*- test
Parental treatment	df = 2		df = 2	
	LR Chisq = 81.225		LR Chisq = 8.538	
	***P*** **<0.001**	Naive – PBS: ***P<0.001***	***P*** **<0.05**	
		Naive – PGN: ***P<0.001***		
		PBS – PGN: *P* = 0.195		
Offspring stage	df = 5		df = 6	
	LR Chisq = 34.131		LR Chisq = 118.352	
	***P*** **<0.001**	Table S10 in File S1	***P*** **<0.001**	
Parental treatment x	df = 10		df = 12	
Offspring stage	LR Chisq = 14.711		LR Chisq = 92.243	
	*P* = 0.143		***P*** **<0.001**	Table S11 in File S1

Statistical evaluation (Generalized linear model; post hoc *U*-test) of priming effects on the change of weight and developmental time of (unchallenged) *Manduca sexta* offspring due to parental treatment (naive, PBS, PGN) (Fig. 4A). Means ± SE are shown in Table S9 in File S1. Significant *P*-levels are shown in bold.

In contrast, the impact of the parental treatment on offspring developmental time were significantly dependent on the ontogenetic offspring stage (Table 4, factor parental treatment × offspring stage, *P*<0.001, Table S9, absolute data in File S1). Young larvae (2^nd^ and 3^rd^ instar) and the wandering larval stage derived from PGN-treated parents showed a significantly shorter developmental time than larvae of untreated parents, whereas the L5 stage of PGN-treated parents showed a longer developmental time than those of untreated parents (Table S11, post-hoc Tukey test in File S1). The duration of the pupal and adult stage of offspring derived from PGN treated parents did not significantly differ from the duration of the respective stages derived from untreated parents (Table S11 in File S1).

### Fecundity

**Figure 5:**
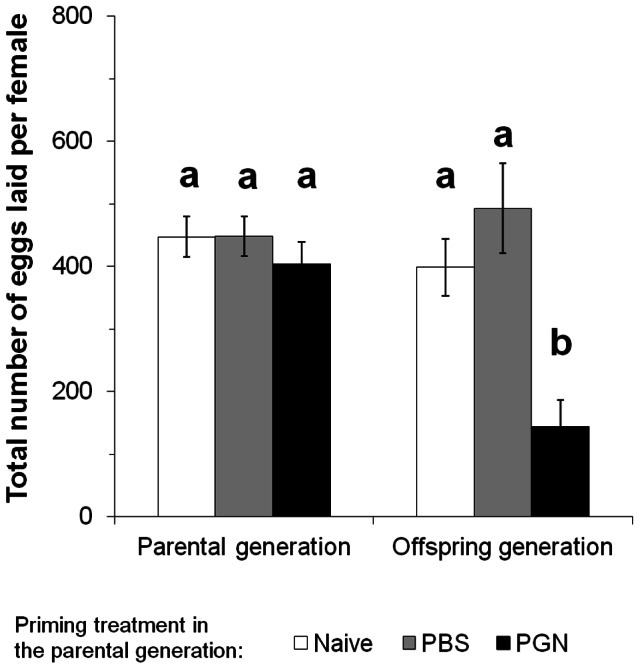
Reproductive fitness of *Manduca sexta* females in the parental and offspring generation. Total number of eggs laid by females of the differently treated parental generations and by the resulting (untreated) offspring generation. Female and male parents received a priming treatment in their pupal stage: Naive) untreated, PBS) control-injected with phosphate buffered saline, PGN) injected with peptidoglycan. Untreated offspring females mated with untreated males that were originating from parents subjected to the same parental priming treatment. Means ± SE are given. *N = *8 individuals from each parental group. Statistics: 2-way-ANOVA, post hoc analysis Tukey-test (Table 5), Tukey-test: different letters indicate statistical differences (*P*<0.01).

The effects of the parental immune treatment on fecundity of adults were dependent on the generation studied (Table 5, two-way ANOVA, parental treatment × generation factor, *P*<0.001). Treatments of the parental generation in the pupal stage with PBS or PGN had no effect on the fecundity of adults in the parental generation (Fig. 5). However, the parental priming treatment significantly affected fecundity of unchallenged offspring. Mated offspring females of PGN-treated parents laid only about a quarter of the eggs compared to the number of eggs laid by offspring females of control parents (Fig. 5).

**Table 5:** Fecundity of parental and unchallenged offspring females.

Source	df	MS	*F*	*P*
Parental treatment	2	168728	10.26	**<0.001**
Generation	1	92225	5.61	**<0.05**
Parental treatment × Generation	2	96913	5.89	**<0.001**

Statistical evaluation (two-way ANOVA) of reproductive fitness of *Manduca sexta* females after immune treatment (naive, PBS, and PGN) of the parental generation and of their unchallenged offspring (Fig. 5). Significant *P*-levels are shown in bold.

## Discussion

Our study showed that a parental immune challenge of *M. sexta* with PGN had different effects on the immune activity and performance of the various offspring life stages when these were kept unchallenged. In contrast, TGIP effects on the immune activity of immunochallenged offspring did not vary among the offspring stages. TGIP also affected the persistence of increased immune activity of offspring that was immunochallenged. The various TGIP effects on immune activities of the offspring life stages were also dependent on the immune parameter studied, i.e. PO activity and antibacterial activity.

### TGIP Effects on Immunity of Unchallenged Offspring

PO activity of the different unchallenged offspring stages was differently affected by TGIP (Fig. 1A, Table 1). A TGIP effect on PO activity was traceable until the 4^th^ instar of unchallenged offspring larvae of PGN- and PBS-challenged parents when compared to offspring larvae of untreated control parents, indicating that the enhanced PO activity in the primed larvae was due to the parental experience of an injection treatment and thus, a slight injury rather than to exposure to pathogens or bacterial surface molecules like PGN (Fig. 1A, Table S3 in File S1). PO is well known to be released from specialised haemolymph cells in response to physical injury; several studies strongly indicate that PO plays a role in haemolymph coagulation and thus in wound healing [42]. Interestingly, PO activity was not enhanced in the parents after injection of PBS or PGN into the fully sclerotized 21-day-old pupae (Table S1 in File S1), but only in their non-challenged larval offspring. This result suggests that the immune system of offspring larvae with their soft bodies can be prepared for a physical injury by an experience of just a slight wounding in the parental pupal stage. However, a study of the moth *Trichoplusia ni* has shown that an increase of PO activity may also occur in larval offspring when the parents had not been challenged by wounding, but were exposed to dietary bacteria [16]. PO-generated reactive compounds may contribute to intoxication of bacteria and fungi [39]; hence, an increased cytotoxic defence may help cope better with bacteria and fungi in an unpredictable environment.

We could not detect a TGIP effect on antibacterial activity of the unchallenged offspring stages of *M. sexta* studied here (Fig. 1B, Table 1), even though antibacterial activity of the parental pupae and adults were significantly enhanced after the PGN immune challenge in the parental pupal stage (Table S1 in File S1). Once produced, AMPs may persist for some time since their highly compact structure hampers access of proteases [43]. This may explain maintenance of high AMP activity in parental adults after treatment in the pupal stage. However, the lack of a TGIP effect on the antibacterial activity of unchallenged offspring larvae may be due to the diverging risks that are experienced by adults and larvae. Larvae usually leave their natal site and thus, are exposed to other risks than the parents. Hence, as long as larvae do not face a bacterial challenge, TGIP of antibacterial activity of unchallenged offspring stages of *M. sexta* might be too costly when considering the unpredictability of the disease that will be experienced by the offspring. However, as soon as offspring larvae need to cope with a bacterial challenge, they appear to be “prepared” for this challenge if their parents have experienced an immune challenge (see Fig. 2B; and below “TGIP effects on immunity of challenged offspring”).

In contrast to our findings, antibacterial activity of non-challenged larvae of the philopatric insect *Tenebrio molitor* was enhanced when their parents experienced a bacterial challenge by injection of lipopolysaccharides (LPS) in the larval stage. Larval *T. molitor* PO activity was not affected by the parental immune challenge [15]. Different *T. molitor* generations may spend their development in the same, stable environment (e.g. in a mill), and therefore they might encounter very similar risks. Thus, it might be beneficial if the immune challenge experienced by the parental generation primes an immunological trait that addresses the likely risks that will also be experienced by the offspring. Similarly, TGIP effects on offspring immunity that were found in the bumblebee *Bombus terrestris* have been suggested to be adjusted to the risk experienced by the unchallenged offspring. A bacterial challenge of *B. terrestris* queens by injection of LPS enhanced the PO activity of unchallenged offspring males that leave the colony and are likely to encounter different pathogens [43]. In contrast, unchallenged females that remain in the nest and encounter the same disease environment as the queen showed an enhanced antibacterial activity which provides a narrower immunity than the one provided by the PO system [43].

### TGIP Effects on Immunity of Challenged Offspring

TGIP effects on PO and antibacterial activity of PGN-challenged offspring did not significantly depend on the developmental offspring stage studied (Fig. 2A, B, Table 2). In contrast to the TGIP effect on PO activity of unchallenged *M. sexta*, no such effect on PO activity was detectable anymore when offspring larvae experienced an immune challenge by PGN injection.

However, TGIP affected the antibacterial activity of the PGN-challenged offspring; the effect was traceable until the pupal stage of the offspring from PGN parents. Both the offspring larvae and pupae with PGN parents showed enhanced antibacterial activity one day after PGN challenge, i.e. after experiencing the risk of bacterial infection. Hence, TGIP effects on PGN-induced antibacterial activity did not differ between offspring larvae and pupae.

### TGIP Effects on Persistence of PGN-challenged Offspring Immunity

The TGIP effects on PGN-induced offspring antibacterial activity were dependent on the time past offspring challenge (Fig. 3B, Table 3). In contrast to offspring derived from control parents, 3-day-old adults from PGN-treated parents were unable to maintain an induced level of antibacterial activity five days after the PGN-treatment in the pupal stage (Table S7 in File S1). The duration of increased antibacterial activity in insect haemolymph ranges between days and weeks (summarized in [41]). Our results show that the parental immune legacy may shorten the duration of increased immune activity in the offspring. Innate immunity and metabolism of insects has been shown to be closely linked, and the production of antimicrobial peptides depends on the energy status of the cells [44]. The resources for mounting enhanced immune activity in the offspring adults derived from PGN-treated parents might have been exhausted after (i) deployment of increased inducibility of antibacterial activity in response to a PGN-challenge in the pupal stage compared to pupae of control parents (Fig. 2B) and/or (ii) maintenance of enhanced immune activity throughout the entire juvenile development (Fig. 1).

### Developmental Stage and Offspring Immunity

Independent of TGIP, immune activity measured as PO and antibacterial activity increased in the offspring generation from the second larval stage to the prepupal stage (Fig. 1). These findings reflect the patterns of susceptibility of insects to parasitoids and pathogens; young insect larvae usually show a higher risk of being parasitized than older ones and are less resistant against parasitism [45]; furthermore, the susceptibility to pathogen infection often decreases with increasing larval stage [46]. While in our study offspring immune activities increased about 2- to 3-fold in response to a parental immune challenge, offspring immune activities increased more than 10-fold during larval development regardless of the parental immune state. Hence, the developmental stage has an enormous impact on the level of immune activity.

The decrease of immune activities in the pupal and adult stage (Fig. 1) might be explained by the formation of a hard exoskeleton in the pupae. The hard cuticle is usually regarded as an effective first barrier against pathogens and parasites [47] and thus, may take on an important task of defence. After the pupal stage, PO activity increased again in the unchallenged offspring adults, which might be due to the high risk of wounding for the moths when actively flying around searching for mates or oviposition sites. In contrast, antibacterial activity of unchallenged offspring adults stayed at a low level, but could easily be induced when it was needed (after PGN-challenge; Fig. 3B). As the PO cascade provides a broader immunity and can be induced more quickly than the costly antibacterial immune response [47], adults may benefit from keeping the most general means of immune defence (the PO system) at an enhanced level. Adult honeybees showed an increasing level of PO activity with increasing age and a decreasing number of haemocytes the older they are [48]; a programmed change in immune functions, from cellular-based to PO-based immunity was observed in the course of ageing of adult bees. Variation of non-primed immune activity across developmental stages of other insects than *M. sexta* has been shown in several studies [23], [49]–[51]. All studies showed that the immune state of the different life stages change in the course of the individuaĺs ontogenesis. The stage-specific immune activities may be adapted to the stage-specific needs of the respective species.

### TGIP Effects on Performance of Unchallenged Offspring

TGIP effects on offspring performance were dependent on the ontogenetic offspring stage when considering developmental time of the offspring as performance parameter (Fig. 4B, Table 4). In the field, *M. sexta* suffers a high mortality (approx. 90%) caused by parasitoids and predators which mainly attack the young larval stages [26]–[28]. A field study with *M. sexta* showed that rapid larval development was associated with higher survival to pupation and thus, was suggested to allow larvae to escape from attack by natural enemies [28]. Therefore, a shorter developmental time of 2^nd^ and 3^rd^ instar larvae in offspring of PGN-parents – as was found here (Table S11 in File S1) - may be beneficial, since this lowers the high predation and parasitisation risk of these stages.

TGIP effects on offspring performance did not depend on the offspring developmental stage when considering offspring weight (Table 4). Almost all stages derived from parents that experienced PBS- or PGN-injection gained benefit from parental immune priming in terms of higher weight. The parental treatment effects on larval weight and developmental time found here were detected when feeding larvae ad libitum with artificial diet. Whether TGIP would still affect offspring weight and developmental time when larvae would be provided with limited food resources – as it might occur in the field – remains to be investigated by future studies.

The positive TGIP effects on *M. sexta* weight and developmental time contrast other studies of other insect species which showed worse performance of offspring of immunochallenged parents [16], [21], [22]. However, in our study, the benefits that especially young larval *M. sexta* stages gained when their parents had been immunochallenged were at the cost of fecundity of offspring adults. Heavier offspring females of PGN-challenged parents laid unexpectedly fewer eggs than lighter offspring females of control parents (Fig. 4A, 5). Heavy pupae with high fecundity [28] are usually expected to contain large ovaries and more eggs than smaller pupae. In our study, the heavy pupae of PGN-parents may have been rather filled with a large fat body that produced antimicrobial peptides [47] than with large ovaries. Reduced fecundity of offspring adults was found in *Tribolium castaneum* offspring that derived from fathers that experienced an immune challenge, whereas an immune challenge of the mother had no effect on the fecundity of the adult offspring [21]. We did not differentiate between maternal and paternal TGIP effects since we focused on the elucidation of TGIP effects on the various offspring stages rather than on the maternal and paternal contribution to TGIP. If in nature transgenerationally primed *M. sexta* offspring individuals will show a higher survival rate because of faster development and higher level of immunocompetence when facing pathogen infection and parasitoid attack during their juvenile development, more offspring females would be able to lay eggs and to establish the next generation. Such a higher number of offspring females derived from PGN-treated parents might balance their lower fecundity when considering population growth.

### Conclusions

Our results show that the different ontogenetic offspring stages of *M. sexta* respond stage-specifically to the parental immune legacy. The parental immune challenge primes PO activity of their unchallenged young larvae (L2 to L4) which are in the field exposed to a high risk of parasitism and predation; TGIP further leads to a reduction of the developmental time of these stages and thus, provides the young offspring with traits that might improve their ability to cope with carnivorous enemies. The parental immune challenge further primes antibacterial activity of PGN-challenged larvae and pupae which are exposed to the risk of pathogenous infection especially in the pupal stage when digging into the soil. However, our data indicate that the adult offspring needs to pay for the benefits that they gain during juvenile development from TGIP. When exposed to an immune challenge, adult offspring of immunochallenged parents showed a reduced ability to maintain high antibacterial activity for a longer time; furthermore, offspring adults of immunochallenged parents showed reduced fecundity. We suggest that the TGIP effects are adapted to the needs and the risks of each offspring developmental stage.

Overall, our study revealed that TGIP effects on the offspring strongly depend on the ontogenetic offspring stage studied, the immune and performance parameter that is considered and the time past offspring immune challenge. There is growing evidence that insect immune responses show specificity with respect to the pathogen that is faced [52], [53]. Furthermore, the food, gut microbiota, and social interactions may affect insect immunity [4]. Hence, the effects of TGIP on offspring immunity are shaped by a multifaceted range of factors and thus, immunoprimed offspring individuals may be able to fine-tune their immune responses to parasitoids and pathogens with respect to their age, their risks and the prevailing environmental conditions.

## Materials and Methods

### Insect Culturing

*Manduca sexta* were reared in the laboratory at 24°C, 70% r.h., and a 16∶8 h L:D cycle. Larvae were fed ad libitum on a wheat germ based diet (240 g wheat germ, 50 g casein, 35 g agar, 16 g Wesson salt mix, 8 g ascorbic acid, 4 g sorbic acid, 2 g methyl-p-hydroxybenzoate, 20 mg nicotinic acid, 10 mg riboflavin, 4.7 mg thiamin, 4.7 mg pyroxidine, 4.7 mg folic acid, 0.4 mg biotin, and 40 mL 4% formaldehyde per 1.2 L water). Eggs, larvae and pupae were kept in boxes of different sizes (approx. 100 eggs, 100 L1 or 50 L2 instars in 20×20×6 cm boxes; approx. 30 L3 or 30 L4 instars in 21×36×13 cm boxes; 10–20 L5 instars in 26×41×15 cm boxes; 3–5 wandering L5 instars or 10 pupae separated by sexes in 13×17×6 cm boxes). Each developmental stage was always available in the rearing. Eclosing adults were allowed to mate inside flight cages (50×50×50 cm, approx. 6 females and 6 males per cage) and were provided with 10% aqueous honey solution and a tobacco leaf for stimulation of oviposition. The tobacco leaf was placed on the top of a glass jar (250 mL) that was wrapped with parafilm. The parafilm also fixed the margins of the leaf to the jar. Moths preferred to lay eggs on the parafilm while landing on the tobacco leaf and then curling their abdomen to the parafilm-wrapped glass jar. Eggs could easily be removed from the parafilm and were used for further rearing. The leaf was replaced every other day by a fresh one taken from tobacco plants (about 6 weeks old) that were grown in the greenhouse.

### Priming Treatment in the Parental Generation

The parental and offspring generation were reared at the same abiotic conditions as described above.

For the priming treatment of the parental generation, we used *M. sexta* pupae 21 days after pupation (Table S1, S2 in File S1). Female pupae of this age always showed completely developed ovaries (observed by dissection of 14- to 21-day-old pupae). The priming treatment of the parental generation was conducted in pupae of this age, since we wanted to expose the parental generation to an immune challenge just prior to egg maturation, i.e. just prior to the onset of formation of the next generation. The first mature eggs were found in *M. sexta* ovaries 24 h after eclosion of adults [54]. Both male and female pupae of the parental generation were subjected to an injection of peptidoglycan extracted from *Micrococcus luteus* (PGN, Sigma 53243). For control, males and females were injected a phosphate buffered saline (PBS; 7 mM KH_2_PO_4_, 3 mM Na_2_HPO_4_, 0.13 M NaCl, pH 7.4) or were left untreated (no challenge, naive). Hence, three different parental groups were generated.

Disposable 1 mL polypropylene insulin syringes (BD Consumer Healthcare, Franklin Lakes, USA) were used for injections. Each PBS-treated pupa received an injection of 50 µL PBS solution dorsally into the first abdominal segment after being chilled on ice. Each PGN-challenged pupa received a 2 µg µL^−1^ dose of PGN extracted from *M. luteus* in 50 µL sterile PBS solution after being chilled on ice.

When referring to adult parents, these were only adults which eclosed 2 days after pupal treatment, and which were in the adult stage for 1 or 3 days (see below). Since an individual’s immune response is dynamic over time [40], [55], 3-day-old adults of each parental group were used for production of the offspring generation. After eclosure, adults were kept in cages (50×50×50 cm; four females and four males of the same priming treatment per cage). The immune priming treatment of the parental generation by PGN injection led to an increase of antibacterial activity in the parental pupae and adults (data shown in File S1, Table S1, S2).

The offspring generation was established by using only eggs laid by 3-day-old females, i.e. - in the case of offspring derived from treated parents - five days after the parental priming treatment in the pupal stage. Thus, we could exclude that variation in offspring immune responses were due to different time intervals between parental treatment and egg deposition. Different time intervals between these events were shown to affect offspring immunity [22].

### Offspring Culturing and Treatment

Eggs laid by different females originating from different generations were taken to rear the offspring used for the experiments. Freshly laid eggs of each parental group were taken from the oviposition jar in a cage and placed on artificial diet (see above) in small plastic cups (diameter 8 cm, height 6 cm, five eggs per cup). Larvae were maintained in these cups with five larvae per cup until the 4^th^ instar, whereas 5^th^ instar larvae were kept in larger boxes with three larvae per box (13×17×6 cm) which were cleaned and filled with fresh artificial diet every other day. Keeping the larvae at these densities ensured avoidance of density-dependent effects on immunity [56]. When larvae reached the wandering stage, they were kept further on in the larger boxes, but were no longer provided with diet; the boxes were only lined with household paper. Pupae were separated by sexes, and always 10 pupae were kept in the larger boxes between two layers of household paper. Adults eclosed in 50×50×50 cm cages and were provided with 10% aqueous honey solution. None of the offspring individuals used for immunity measurements was returned to the experimental rearing.

In order to examine the impact of the parental priming treatment on *M. sexta* offspring immunity, (i) immunity of different unchallenged offspring life stages (2^nd^ –5^th^ instar larvae, wandering larvae, pupae, and adults) (Fig. 1, Table 1, Table S3, S4 in File S1) and (ii) immune responses of challenged 4^th^ instar larvae and pupae of naive, PBS- and PGN-treated parents were compared (Fig. 2, Table 2, Table S5, S6 in File S1). Offspring larvae, pupae and adults from each parental group were sampled at the first day of each developmental stage in order to measure immune parameters of unchallenged offspring. In addition, unchallenged 22-day-old offspring pupae of each parental treatment group were sampled for immunity measurements of unchallenged offspring.

The offspring immune system was challenged by PGN injection. For control, offspring larvae and pupae received a PBS injection or were left untreated (naive). Larvae (4^th^ instar) were treated at the day of molting, and haemolymph was sampled one day later. Nine offspring larvae were sampled at random from each parental group and received a single injection of either 25 µL PGN solution (2 µg PGN/µL PBS) or 25 µL PBS solution through the ‘horn’ on the terminal abdominal segment. The PGN- and PBS-treatments of 21-day-old pupae were conducted in the same way as described above for the parental generation.

In order to investigate the impact of the parental priming treatment on the time for which a once enhanced immune activity level persists after immune challenge in the offspring, haemolymph was sampled three days (in 1-day-old adults), and five days (in 3-day-old adults) after treatment of offspring pupae from each parental group (Fig. 3, Table 3, Table S7, S8 in File S1). At each time point nine offspring individuals were sampled at random for each offspring immune treatment (naive, PBS, PGN; i.e. in total, 162 individuals were analysed; 3 immune treatments for offspring pupae, 3 parental groups, 2 measurement time points, 9 offspring adults per group: 3×3×2×9 = 162).

### Sampling Haemolymph

All individuals were surface-sterilised with a 70% ethanol-soaked paper towel prior to handling. We determined the immune activity of haemolymph taken from chilled larvae, pupae and adults. Larval haemolymph was removed from an incision made in the ‘horn’ with scissors; wounding of other developmental stages was conducted with a scalpel. Haemolymph of wandering larvae was taken from a wound inflicted to the last proleg. Pupal haemolymph was collected from a dorsal cut between thorax and abdomen. Adult haemolymph was taken from a ventral cut between the second and third thoracic segment of which hairs were removed. In pupae, we first differentiated between male and female haemolymph samples. Pilot experiments revealed that this *M. sexta* stage which we challenged in the parental generation did not show sexual dimorphism in immune responses (Table S2 in File S1). Hence, we did not separate haemolymph samples of pupae and adults according to sex in our further experiments since our studies focussed on the impact of parental priming on the different offspring developmental stages rather than on the elucidation of sex-specific TGIP effects. Haemolymph that emerged from the experimentally inflicted injuries of larvae (different instars at the day of molting), wandering larvae (at the day of aorta exposure), pupae (1- and 22-day-old) and adults (1- and 3-day-old) was sucked with a 10 µL-pipet into pipet tips (L2∶1 µL, L3∶2 µL, L4– adults: 6–12 µL per individual). We did not collect haemolymph of L1 larvae since they are too small for haemolymph sampling. Nine samples were collected for each developmental stage and treatment. To obtain enough haemolymph for the assays, one sample of L2 haemolymph consisted of haemolymph that was pooled from six individuals, and one sample of L3 haemolymph was pooled from three individuals, whereas each sample of haemolymph of other stages was obtained from a single individual. Haemolymph samples were immediately diluted 1∶5 in anticoagulant buffer (4 mM NaCl, 40 mM KCl, 4 mM EDTA, 4.8 mM citric acid, 13.6 mM sodium citrate, 5% saccharose, 0.1% polyvinylpyrrolidone, 1.9 mM PIPES, pH 6.8), frozen in liquid nitrogen, and stored at −80°C until used for analysis.

### Immune Parameters

As proxy for the activity of the immune status of parental and offspring stages we measured phenoloxidase (PO) and antibacterial activity of the haemolymph of juvenile and adult stages. Samples for analyses of PO and antibacterial activity were kept frozen and thawed on ice directly prior to analysis.

- **Phenoloxidase (PO) activity.** A haemolymph sample (5 µL) was diluted 1∶2 in Na_2_HPO_4_ and used for a dot blot assay as described by [57], but modified as follows: instead of 10 mM Na_2_HPO_4_, we used a 1 mM concentration of this buffer; a dilution series (200.0, 100.0, 50.0, 20.0, 10.0, 5.0, 2.5, 1.7, 1.25, 1×10^−3^) of fountain pen ink (Pelikan 4001 brillant black, Hannover, Germany) was used to establish a calibration curve (formular of calibration: value of measured darkness = 12.7ln(ink dilution) –96.8 to compute the melanisation index [ink dilution×10^−3^]); dry filterpapers were scanned with AlphaImager® (Alpha Innotech, Kasendorf, Germany). A control of each sample on a filter paper pre-soaked with 1 mM Na_2_HPO_4_ was used to measure the darkness and the spontaneous PO activity of the samples. The value of the control measurement without L-Dopa was subtracted from the L-Dopa measurement to calculate the value of measured darkness. Data are presented as melanisation index per individual. Hence, measurements of L2 haemolymph were always divided by 6, and those of L3 haemolymph were always divided by 3 (compare above, haemolymph sampling).
- **Antibacterial activity.** A haemolymph sample (5 µL) was diluted 1∶4 in PBS before measurement. Test plates (12×12 cm) were prepared by adding 2.5 mL of PBS-washed *M. luteus* (DSM 20030, DSMZ, Braunschweig, Germany) bacteria suspension (OD620 = 0.6) to 25 mL sterile medium (5 g yeast extract, 5 g pepton in 1 L distilled water, pH 7.0, 1.5% agar). A cork borer was used to punch 25 wells in one agar plate. A volume of 20 µL of each sample solution was added to each well. Plates were incubated for 24 h at 30°C. A dilution series of chicken lysozyme (Sigma; 500.0, 250.0, 125.0, 62.5, 31.3, and 15.6 µg mL^−1^) was applied to each plate to generate a calibration curve based on these standards. Antibacterial activities were determined as lysozyme equivalents calculated from the radius of the clear zone around a sample well. Data are given in lysozyme equivalents per individual; hence, data of L2 haemolymph were always divided by 6, and data of L3 haemolymph were always divided by 3 (compare above, haemolymph sampling).

Increase of PO or antibacterial immune activity in the challenged larval, pupal and adult offspring was analysed for each parental group by comparing the activity elicited by PBS or PGN treatment with the activity in the haemolymph of unchallenged individuals (Fig. 2). We calculated for each developmental stage studied:
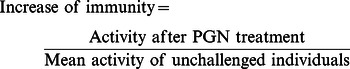



Data that were used to calculate the increase are shown in Table S5 in File S1. Increase calculation allows a comparison between the immune activities of the different immunochallenged developmental stages.

### Offspring Weight, Developmental Time and Fecundity

In order to elucidate benefits and costs of the parental priming treatment in course of the development of the offspring generation, we recorded the following parameters: weight of each stage from 2^nd^ instar to adult, developmental time of each life stage from egg to adult (Fig. 4, Table 4, Table S9, S10, S11 in File S1), and the number of eggs that were laid per female in the parental and the offspring generation (Fig. 5, Table 5).

Eighteen eggs of each parental group were taken from the oviposition jars in the cages with the respective parents. Each egg was placed individually in a small cup (diameter 8 cm, height 6 cm) on artificial diet (see above) under the abiotic conditions used for *M. sexta* rearing. Larvae were fed ad libitum on artificial diet. The 5^th^ instar larvae and wandering larvae were kept in larger boxes (13×17×6 cm). Larval weight was recorded at the last day of each instar. Pupae and adults were weighed at the first day of the respective life stage. To record fecundity, offspring females were kept individually with one male derived from the same parental treatment at the conditions as described above for the *M. sexta* rearing.

To compare weight and developmental time of offspring derived from naive parents with offspring from parents that received a priming treatment we calculated the ratios of weight or developmental time between offspring from naive parents and PBS- or PGN-treated parents:
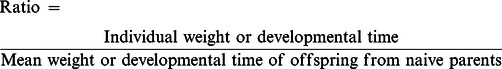



Data that were used to calculate these ratios are shown in Table S9 in File S1. Ratio calculation allows a comparison between the performance parameter of the different developmental stages.

### Statistics

All statistical analyses were conducted with R 2.12.0 (R Developmental Core Team, 2010). Data were analysed using descriptive statistics and were tested for normality by Shapiro-Wilk test and for variance homogeneity by Levene test. The data were Box-Cox transformed to reach normal distribution if necessary.

To determine whether a parental immune challenge affected the immunity of unchallenged offspring individuals differently in the different offspring developmental stages, PO and antibacterial activities of the offspring were analysed by a two-way-ANOVA (factors: (i) parental treatments: naive, PBS, PGN and (ii) offspring stage: 2^nd^, 3^rd^, 4^th^, 5^th^ larval instar, wandering larvae, 1-day- and 22-day-old pupae, 1-day-old adults (Table 1 for ANOVA, Fig. 1)). Finally, a post-hoc Tukey test was performed to compare the different developmental stages of the three parental treatment groups stage by stage (Table S3 for PO activity in File S1) or to compare the different developmental stages (Table S4 for antibacterial activity in File S1).

The effects of an immune challenge of offspring individuals on immune activities were expressed as increase of immunity in immunochallenged offspring individuals when compared to untreated ones (see more detailed explanation above). In order to determine whether the different parental treatments affected the immunity of PGN-immunochallenged offspring individuals differently with respect to the offspring developmental stage that was challenged, data were analysed by using a two-way-ANOVA (factor: (i) parental treatments: naive, PBS, PGN and (ii) offspring stage: 4^th^ larval instar, 22-day-old pupae). Fig. 2 and Table 2 (ANOVA) show the results obtained from the comparison of PGN-challenged offspring of the three parental treatment groups. Table S5 in File S1 presents the absolute data (no increase data). For control, Table S6 (ANOVA) in File S1 shows the results obtained from the comparison of PBS-treated offspring of the three parental treatment groups with respect to the question whether the different parental treatments affected the immunity of PBS-treated offspring individuals differently.

In order to elucidate whether the parental treatment affects the persistence of immune activity of the PGN-treated offspring, data were analysed by using a two-way-ANOVA (factor: (i) parental treatments: naive, PBS, PGN and (ii) time after immune offspring immune challenge: 3 days (1-day-old adults), 5 days (3-day-old adults)). Fig. 3 and Table 3 (ANOVA) show the results obtained from the comparison of PGN-challenged offspring of the three parental treatment groups. Table S5 in File S1 presents the absolute data (no increase data). The antibacterial activities of challenged offspring adults of the different parental treatment groups were compared by a post-hoc Tukey test (Table S7 in File S1). For control, Table S8 in File S1 shows the results obtained from the comparison of PBS-treated offspring of the three parental treatment groups with respect to question for how long the parental treatment effects on the immunity of pupae persist after pupal PBS treatment.

The effects of the parental immune treatment on offspring weight and development time were expressed as ratio of the performance parameter of offspring from PGN (PBS)-challenged parents and untreated parents (see more detailed explanation above). The ratio expresses the increase or decrease in performance in dependence of the parental treatment. In order to determine whether the different parental treatments affected the weight or developmental time of the offspring individuals differently with respect to the offspring developmental stage, data were analysed by using a generalized linear model (GLM) since the data did not show homogeneity of variance nor normal distribution (factor: (i) parental treatments: naive, PBS, PGN and (ii) offspring stage: 2^nd^, 3^rd^, 4^th^, 5^th^ larval instar, 1-day-old pupae, 1-day-old adults). Fig. 4A, B and Table 4 (GLM) show the results obtained from the comparison of the offspring of the three parental treatment groups. Table S9 in File S1 presents the absolute data (no ratios). The weights of the different offspring stages were compared by post-hoc *U* tests with Benjamini-Hochberg correction (Table S10 in File S1). The ratios of developmental time of the different developmental stages of the three parental treatment groups were compared stage by stage by a post- hoc *U*-test with Benjamini-Hochberg correction (Table S11 in File S1).

In order to determine whether fecundity in the parental and the offspring generation changed due to the parental priming treatment, data were analysed using a two-way-ANOVA with parental treatments (naive, PBS, PGN) and generation (parental generation and offspring generation) as factors (Table 5, Fig. 5). Finally, a Tukey test was performed to compare means (Fig. 5).

## Supporting Information

File S1 (DOCX)
