# Supplementary material for: Parental Legacy in Insects: Variation of Transgenerational Immune Priming during Offspring Development
Source: PLoS One. 2013 May 20;8(5):e63392. doi: 10.1371/journal.pone.0063392 (PMC3658988; doi:10.1371/journal.pone.0063392)
Supplement: File S1 — (DOCX) [file pone.0063392.s001.docx]

**Online Supporting Information File S1**

**Parental legacy in insects:**

**Variation of transgenerational immune priming**

**during offspring development**

Ute Trauer and Monika Hilker

Institute of Biology – Applied Zoology/Animal Ecology, Freie Universität Berlin,
Berlin, Germany

*Parental priming treatment*

The immune priming treatment of the parental generation by PGN injection into 21-day-old pupae led to a significant increase of antibacterial activity in 22-day-old pupae, i.e. one day after priming treatment when compared to untreated or buffer (PBS) control-injected pupae (Table S1). We did not detect any sex specific antibacterial responses by the pupae to the PGN treatment (Table S2). The antibacterial activity in the parental pupae was persistent for at least five days after priming treatment, i.e. it was detectable also in 3-day-old adults (Table S1). Hence, antibacterial activity was still induced in PGN-treated females at the time point when they laid the eggs that were used to produce the offspring generation for our studies. In contrast, phenoloxidase (PO) activity of the parental generation was not significantly affected by the PGN treatment (Table S1). Again, no sex specific differences in PO activities were detected between PGN treated male and female pupae (Table S2). A control treatment by buffer (PBS) injection into the parental pupae did neither lead to an increase of PO activity nor of antibacterial activity in the parental generation when compared to untreated (naive) parents (Table S1).

**Table S1.** Parental generation. Statistical evaluation of immune activity (phenoloxidase and antibacterial activity / lysozyme activity equivalents, *Micrococcus luteus*; means ± SE of absolute data) of *Manduca sexta* parents (males and females) one day after immune treatment (22-days-old pupae), three days after treatment (1-day-old adults), and five days after treatment in the pupal stage (3-day-old adults). Parental treatment in 21-day-old pupae: Naive) untreated, PBS) control-injected with phosphate buffered saline, PGN) injected with peptidoglycan.

|  | **Phenoloxidase activity** | | | | **Antibacterial activity** | | | |
| --- | --- | --- | --- | --- | --- | --- | --- | --- |
|  | Melanisation index^1^ (ink dilution * 10^-3^) | | | | Lysozyme equivalent ^2^ (µg * ml^-1^) | | | |
| **Parental**  **ontogenetic** |  |  |  |  |  |  |  |  |
| **stage** | Naive | PBS-treated | PGN-treated | *P* | Naive | PBS-treated | PGN-treated | *P* |
| **Pupae** |  |  |  |  |  |  |  |  |
| 22 d | 58.8 ± 4.0 | 65.4 ± 11.3 | 61.8 ± 8.8 | n.s. | 12.8 ± 2.1 **^a^** | 13.6 ± 2.7 **^a^** | 65.4 ± 9.5 **^b^** | *** |
| **Adults** |  |  |  |  |  |  |  |  |
| 1 d | 82.4 ± 23.4 | 98.9 ± 16.5 | 103.5 ± 23.2 | n.s. | 13.4 ± 1.9 **^a^** | 14.5 ± 3.8 **^a^** | 60.0 ± 5.3 **^b^** | *** |
| 3 d | 116.5 ± 33.4 | 145.2 ± 15.1 | 118.2 ± 23.4 | n.s. | 7.5 ± 1.3 **^a^** | 11.0 ± 3.6 **^a^** | 31.7 ± 4.8 **^b^** | *** |

^1^ Phenoloxidase activity determined by melanisation index measured as raw pixel value after reaction of haemolymph with 2 mM L-Dopa

^2^ Antibacterial activity measured as the diameter of the lytic zone on agar plates with *Micrococcus luteus* and transformed into lysozyme equivalents (µg ml^-1^).

Statistics: Comparison between different treatments within each developmental stage by Kruskal-Wallis *H*-test (*P*: n.s. = not significant: *P* > 0.05, ***: *P*< 0.001), post hoc analysis Mann-Whitney *U*-test with Benjamini-Hochberg correction, different letters indicate statistical differences (*P* ≤ 0.05). *N* = 9 for each developmental stage.

**Table S2.** Parental generation. Statistical evaluation of sex specific immune activity (phenoloxidase and antibacterial activity / lysozyme activity equivalents, *Micrococcus luteus*; means ± SE of absolute data) of female and male *Manduca sexta* pupae (22 days old) one day after immune treatment: Naive) untreated, PBS) control-injected with phosphate buffered saline, PGN) injected with peptidoglycan.

|  | **Phenoloxidase activity** | | | **Antibacterial activity** | | |
| --- | --- | --- | --- | --- | --- | --- |
|  | Melanisation index^1^ (ink dilution x 10^-3^) | | | Lysozyme equivalent ^2^ (µg mL^-1^) | | |
| **Treatment** | Females | Males | *P* | Females | Males | *P* |
| Naive | 44 ± 7 | 36 ± 6 | n.s. | 16.0 ± 4.0 | 8.0 ± 1.9 | n.s. |
| PBS | 40 ± 10 | 36 ± 12 | n.s. | 13.4 ± 3.5 | 23.8 ± 6.9 | n.s. |
| PGN | 36 ± 9 | 44 ± 9 | n.s. | 102.5 ± 27.2 | 94.1 ± 21.9 | n.s. |

^1^ Phenoloxidase activity determined by melanisation index measured as raw pixel value after reaction of haemolymph with 2 mM L-Dopa

^2^ Antibacterial activities measured as the diameter of the lytic zone on agar plates with *Micrococcus luteus* and transformed into lysozyme equivalents (µg mL^-1^).

Statistics: Comparison of females and males within each treatment by Mann-Whitney *U*-test (*P*: n.s. = not significant: *P* > 0.05). *N* = 10 for each gender and treatment.

**Table S3.** Unchallenged offspring generation. Results of a post-hoc Tukey-test which analysed the differences in PO activity of unchallenged *Manduca sexta* between the different offspring stages and different parental treatments (naive, PBS, PGN) (Fig. 1A, Table 1, significant parental treatment x offspring stage factor)

|  |  | L2 | L3 | L4 | L5 | W | 1P | 22P | A | L2 | L3 | L4 | L5 | W | 1P | 22P | A | L2 | L3 | L4 | L5 | W | 1P | 22P | A |
| --- | --- | --- | --- | --- | --- | --- | --- | --- | --- | --- | --- | --- | --- | --- | --- | --- | --- | --- | --- | --- | --- | --- | --- | --- | --- |
|  |  | N | N | N | N | N | N | N | N | PBS | PBS | PBS | PBS | PBS | PBS | PBS | PBS | PGN | PGN | PGN | PGN | PGN | PGN | PGN | PGN |
| L2 | N | - |  |  |  |  |  |  |  |  |  |  |  |  |  |  |  |  |  |  |  |  |  |  |  |
| L3 | N | n.s. | - |  |  |  |  |  |  |  |  |  |  |  |  |  |  |  |  |  |  |  |  |  |  |
| L4 | N | *** | n.s. | - |  |  |  |  |  |  |  |  |  |  |  |  |  |  |  |  |  |  |  |  |  |
| L5 | N | *** | *** | ** | - |  |  |  |  |  |  |  |  |  |  |  |  |  |  |  |  |  |  |  |  |
| W | N | *** | *** | *** | n.s. | - |  |  |  |  |  |  |  |  |  |  |  |  |  |  |  |  |  |  |  |
| 1P | N | *** | *** | ** | n.s. | n.s. | - |  |  |  |  |  |  |  |  |  |  |  |  |  |  |  |  |  |  |
| 22P | N | *** | *** | n.s. | n.s. | n.s. | n.s. | - |  |  |  |  |  |  |  |  |  |  |  |  |  |  |  |  |  |
| A | N | *** | *** | *** | n.s. | n.s. | n.s. | n.s. | - |  |  |  |  |  |  |  |  |  |  |  |  |  |  |  |  |
| L2 | PBS | * | n.s. | n.s. | *** | *** | *** | ** | *** | - |  |  |  |  |  |  |  |  |  |  |  |  |  |  |  |
| L3 | PBS | *** | * | n.s. | * | ** | * | n.s. | *** | n.s. | - |  |  |  |  |  |  |  |  |  |  |  |  |  |  |
| L4 | PBS | *** | *** | ** | n.s. | n.s. | n.s. | n.s. | n.s. | *** | * | - |  |  |  |  |  |  |  |  |  |  |  |  |  |
| L5 | PBS | *** | *** | ** | n.s. | n.s. | n.s. | n.s. | n.s. | *** | * | n.s. | - |  |  |  |  |  |  |  |  |  |  |  |  |
| W | PBS | *** | *** | *** | n.s. | n.s. | n.s. | ** | n.s. | *** | *** | n.s. | n.s. | - |  |  |  |  |  |  |  |  |  |  |  |
| 1P | PBS | *** | *** | *** | n.s. | n.s. | n.s. | n.s. | n.s. | *** | *** | n.s. | n.s. | n.s. | - |  |  |  |  |  |  |  |  |  |  |
| 22P | PBS | *** | *** | n.s. | n.s. | n.s. | n.s. | n.s. | n.s. | ** | n.s. | n.s. | n.s. | ** | n.s. | - |  |  |  |  |  |  |  |  |  |
| A | PBS | *** | *** | * | n.s. | n.s. | n.s. | n.s. | n.s. | *** | n.s. | n.s. | n.s. | * | n.s. | n.s. | - |  |  |  |  |  |  |  |  |
| L2 | PGN | * | n.s. | n.s. | *** | *** | *** | ** | *** | n.s. | n.s. | *** | *** | *** | *** | ** | *** | - |  |  |  |  |  |  |  |
| L3 | PGN | *** | ** | n.s. | n.s. | * | n.s. | n.s. | * | n.s. | n.s. | n.s. | n.s. | *** | ** | n.s. | n.s. | n.s. | - |  |  |  |  |  |  |
| L4 | PGN | *** | *** | ** | n.s. | n.s. | n.s. | n.s. | n.s. | *** | * | n.s. | n.s. | n.s. | n.s. | n.s. | n.s. | *** | n.s. | - |  |  |  |  |  |
| L5 | PGN | *** | *** | *** | n.s. | n.s. | n.s. | n.s. | n.s. | *** | *** | n.s. | n.s. | n.s. | n.s. | n.s. | n.s. | *** | ** | n.s. | - |  |  |  |  |
| W | PGN | *** | *** | *** | n.s. | n.s. | n.s. | ** | n.s. | *** | *** | n.s. | n.s. | n.s. | n.s. | ** | n.s. | *** | *** | n.s. | n.s. | - |  |  |  |
| 1P | PGN | *** | *** | ** | n.s. | n.s. | n.s. | n.s. | n.s. | *** | n.s. | n.s. | n.s. | n.s. | n.s. | n.s. | n.s. | *** | n.s. | n.s. | n.s. | n.s. | - |  |  |
| 22P | PGN | *** | *** | n.s. | n.s. | n.s. | n.s. | n.s. | n.s. | n.s. | n.s. | n.s. | n.s. | *** | * | n.s. | n.s. | n.s. | n.s. | n.s. | ** | *** | n.s. | - |  |
| A | PGN | *** | *** | *** | n.s. | n.s. | n.s. | n.s. | n.s. | *** | ** | n.s. | n.s. | n.s. | n.s. | n.s. | n.s. | *** | n.s. | n.s. | n.s. | n.s. | n.s. | n.s. | - |

Offspring developmental stages: L2 – 2^nd^ instar, L3 – 3^rd^ instar, L4 – 4^th^ instar, L5 – 5^th^ instar, W – wandering stage, 1P – 1-day-old pupae, 22P – 22-day-old pupae, A – 1-day-old adults. Parental priming treatment: N – Naive untreated parents, PBS – control injection with PBS, PGN – immune priming with peptidoglycan injection, Statistics: n.s. – not significant *P* > 0.05, * *P* ≤ 0.05, ** *P* < 0.01, *** *P* < 0.001. Shaded area: Same developmental stages in the offspring of differently treated parents

**Table S4.** Unchallenged offspring generation. Results of a post-hoc Tukey-test which analysed the differences in antibacterial activity (lysozyme activity equivalents, *Micrococcus luteus*) of unchallenged *Manduca sexta* offspring between the different developmental stages (Fig. 1B; Table 1, significant offspring stage factor).

|  | L2 | L3 | L4 | L5 | W | 1P | 21P | A |
| --- | --- | --- | --- | --- | --- | --- | --- | --- |
| L2 | - |  |  |  |  |  |  |  |
| L3 | *** | - |  |  |  |  |  |  |
| L4 | *** | *** | - |  |  |  |  |  |
| L5 | *** | *** | *** | - |  |  |  |  |
| W | *** | *** | *** | n.s. | - |  |  |  |
| 1P | *** | *** | *** | n.s. | n.s. | - |  |  |
| 21P | *** | *** | n.s. | *** | *** | *** | - |  |
| A | *** | *** | n.s. | *** | *** | *** | n.s. | - |

Offspring developmental stages: L2 – 2^nd^ instar, L3 – 3^rd^ instar, L4 – 4^th^ instar, L5 – 5^th^ instar, W – wandering stage, 1P – 1-day-old pupae, 22P – 22-day-old pupae, A – 1-day-old adults. Statistics: n.s. – not significant *P* > 0.05, *** *P* < 0.001.

**Table S5.** Unchallenged (naive) and immunochallenged (PBS, PGN) offspring generation. Absolute data of immune activity (phenoloxidase and antibacterial activity / lysozyme activity equivalents, *Micrococcus luteus*; means ± SE) of *Manduca sexta* offspring. Parental and offspring treatment in the larval and pupal stage: Naive) untreated, PBS) control-injected with phosphate buffered saline, PGN) injected with peptidoglycan. Compare Fig. 2 and 3 for increase of immune parameters in PGN-challenged individuals, Table S6, S8 for increase of immune parameters in PBS-treated individuals and Table S7 for respective post hoc Tukey tests.

|  | **Phenoloxidase activity** | | | | **Antibacterial activity** | | | |
| --- | --- | --- | --- | --- | --- | --- | --- | --- |
|  | Melanisation index^1^ (ink dilution * 10^-3^) | | | | Lysozyme equivalent ^2^ (µg * ml^-1^) | | | |
| **Offspring**  **ontogenetic** | Naive | PBS-treated | PGN-treated |  | Naive | PBS-treated | PGN-treated |  |
| **stage** | Parents | Parents | Parents |  | parents | parents | parents |  |
| **Larvae** |  |  |  |  |  |  |  |  |
| L4 (Naive) | 46.8 ± 13.2 | 85.2 ± 20.3 | 80.4 ± 12.1 |  | 30.7 ± 7.9 | 25.5 ± 5.6 | 22.4 ± 3.9 |  |
| L4 (PBS) | 50.8 ± 13.2 | 110.1 ± 7.6 | 139.5 ± 18.0 |  | 38.1 ± 2.8 | 43.4 ± 3.3 | 31.0 ± 2.9 |  |
| L4 (PGN) | 73.8 ± 15.5 | 101.3 ± 13.7 | 124.6 ± 19.8 |  | 173.4 ± 24.6 | 154.1 ± 20.1 | 295.6 ± 37.6 |  |
| **Pupae** |  |  |  |  |  |  |  |  |
| 22 d (Naive) | 50.3 ± 6.4 | 54.3 ± 10.4 | 42.2 ± 15.4 |  | 20.4 ± 2.1 | 22.3 ± 3.0 | 21.9 ± 2.3 |  |
| 22 d (PBS) | 83.4 ± 11.0 | 68.1 ± 11.1 | 103.0 ± 26.6 |  | 22.9 ± 1.8 | 28.7 ± 3.4 | 28.0 ± 2.2 |  |
| 22 d (PGN) | 70.4 ± 9.4 | 67.6 ± 16.2 | 94.0 ± 29.1 |  | 60.8 ± 8.8 | 61.9 ± 5.5 | 104.4 ± 15.4 |  |
| **Adults** |  |  |  |  |  |  |  |  |
| 1 d (Naive) | 101.5 ± 13.0 | 63.4 ± 7.4 | 96.9 ± 22.8 |  | 14.4 ± 2.6 | 15.1 ± 3.6 | 15.8 ± 2.7 |  |
| 1d (PBS) | 102.9 ± 10.0 | 91.4 ± 17.9 | 112.6 ± 13.3 |  | 12.1 ± 2.0 | 16.4 ± 2.8 | 17.6 ± 2.9 |  |
| 1 d (PGN) | 107.2 ± 14.5 | 76.4 ± 12.2 | 84.5 ± 13.1 |  | 59.2 ± 9.9 | 56.2 ± 14.3 | 56.2 ± 8.1 |  |
| 3 d (Naive) | 101.0 ± 10.9 | 112.7 ± 19.2 | 119.4 ± 18.0 |  | 10.3 ± 1.9 | 13.8 ± 4.0 | 11.2 ± 3.1 |  |
| 3 d (PBS) | 103.7 ± 12.5 | 95.7 ± 17.7 | 89.1 ± 11.9 |  | 9.25 ± 1.6 | 10.6 ± 1.6 | 13.9 ± 3.2 |  |
| 3 d (PGN) | 138.6 ± 22.8 | 99.5 ± 20.1 | 94.4 ± 12.7 |  | 32.2 ± 4.2 | 30.3 ± 6.8 | 11.3 ± 1.7 |  |

^1^ Phenoloxidase activity determined by melanisation index measured as raw pixel value after reaction of haemolymph with 2 mM L-Dopa

^2^ Antibacterial activity measured as the diameter of the lytic zone on agar plates with *Micrococcus luteus* and transformed into lysozyme equivalents (µg ml^-1^).

**Table S6.** PBS-treated offspring generation. PBS = phosphate buffered saline. Statistical evaluation (two-way-ANOVA) of priming effects on the change (“increase” values) of phenoloxidase (PO) and antibacterial activity (AMP; lysozyme activity equivalents, *Micrococcus luteus*) of PBS-treated *Manduca sexta* offspring from differently treated parents (naive, PBS, PGN) one day after PBS treatment in 4^th^ instar larvae and 22-day-old pupae. Means ± SE are shown in Table S5. While this table shows data of the PBS-treated offspring, Fig. 2 and Table 2 refer to data of PGN-treated offspring.

|  | **PO activity after** | **Antibacterial activity** |
| --- | --- | --- |
| **Source** | **PBS treatment** | **after PBS treatment** |
| Parental treatment | df = 2 | df =2 |
|  | MS = 0.362 | MS = 0.068 |
|  | F = 2.191 | F = 2.839 |
|  | *P* = 0.123 | *P* = 0.068 |
| Offspring stage | df = 1 | df = 1 |
| (L4, pupae) | MS = 0.214 | MS = 0.083 |
|  | F = 1.296 | F = 3.468 |
|  | *P* = 0.261 | *P* = 0.069 |
| Parental treatment x | df = 2 | df = 2 |
| offspring stage | MS = 0.134 | MS = 0.038 |
|  | F = 0.810 | F = 1.582 |
|  | *P* = 0.451 | *P* = 0.216 |

Data were Box-Cox transformed prior to analysis in order to reach normal distribution PO = PO^0.5, AMP = AMP^ 0.5_._

**Table S7.** PGN-challenged offspring generation. PGN = peptidoglycan. Results of a post-hoc Tukey-test which analysed the differences in antibacterial activity (lysozyme activity equivalents, *Micrococcus luteus*) between the different PGN-treated offspring stages of *Manduca sexta* of differently treated parents (naive, PBS, PGN) and different times past offspring challenge (compare Fig. 3B, Table 3, significant parental treatment x time after challenge stage factor; induced antibacterial activity).

|  |  | 1dA | 3dA | 1dA | 3dA | 1dA | 3dA |
| --- | --- | --- | --- | --- | --- | --- | --- |
|  |  | N | N | PBS | PBS | PGN | PGN |
| 1dA | N | - |  |  |  |  |  |
| 3dA | N | n.s. | - |  |  |  |  |
| 1dA | PBS | n.s. | n.s. | - |  |  |  |
| 3dA | PBS | n.s. | n.s. | n.s. | - |  |  |
| 1dA | PGN | n.s. | n.s. | n.s. | n.s. | - |  |
| 3dA | PGN | *** | ** | ** | * | *** | - |

Offspring developmental stages: 1dA – 1-day-old adults (= 3 days past challenge), 3dA – 3-day-old adults
(= 5 days past challenge). Parental priming treatment: N – Naive untreated parents, PBS – control injection with PBS, PGN – immune priming with peptidoglycan injection, Statistics: n.s. – not significant *P* > 0.05,
* *P* ≤ 0.05, ** *P* < 0.01, *** *P* < 0.001.

**Table S8.** PBS-treated offspring generation. PBS = phosphate buffered saline. Statistical evaluation (two-way-ANOVA) of priming effects on the persistence of change (“increase” values) of phenoloxidase (PO) and antibacterial (AMP) activity (lysozyme activity equivalents, *Micrococcus luteus*) three and five days after *Manduca sexta* offspring pupal PBS treatment (i.e. immune activity measurements of 3-day-old and 5-day-old offspring adults). Means ± SE are shown Table S5. While this table shows data of the PBS-treated offspring, Fig. 3, Table 3 and Table S7 refer to data of PGN-treated offspring.

|  |  | **Persistence of enhanced immune activity** | |
| --- | --- | --- | --- |
|  |  | **after PBS treatment** | |
| **Source** |  | **Phenoloxidase** | **Antibacterial** |
|  |  | **activity** | **activity** |
| Parental treatment |  | df =2 | df =2 |
|  |  | MS = 0.004 | MS = 0.005 |
|  |  | F = 0.0408 | F = 2.120 |
|  |  | *P* = 0.667 | *P* = 0.131 |
| Time after offspring |  | df = 1 | df =1 |
| challenge in pupal stage |  | MS = 0.071 | MS = 0.0005 |
| (3 d, 5 d) |  | F = 6.598 | F = 0.206 |
|  |  | ***P* = 0.014** | *P* = 0.652 |
| Parental treatment x |  | df =2 | df = 2 |
| Time after challenge |  | MS = 0.019 | MS = 0.0004 |
|  |  | F = 1.735 | F = 0.179 |
|  |  | *P* = 0.187 | *P* = 0.837 |

Data were Box-Cox transformed prior to analysis in order to reach normal distribution
PO_persistence_ = PO_persistence_^0.227, AMP_persistence_ = AMP_persistence_^0.076. Significant *P*-levels are shown in bold.

**Table S9.** Unchallenged offspring generation. Absolute data of developmental time and weight (means ± SE) of *Manduca sexta* offspring of differently immunochallenged parents. Treatment of parents: Naive) untreated, PBS) control injected with phosphate buffered saline, PGN) injected with peptidoglycan. Larval weight was determined at the last day of each larval instar. Pupae and adults were weighed at the first day of the life stage. Compare Fig. 4, Table 4 for ratios of weight and developmental time in unchallenged (naive) individuals that are derived from untreated parents and from PBS- or PGN-treated parents. Table S10, S11 for respective post hoc *U*-tests.

|  | **Developmental time per life stage (days)** | | |  | **Weight (mg or *g*)^1^** | | |
| --- | --- | --- | --- | --- | --- | --- | --- |
| **Offspring**  **ontogenetic** | **Naive** | **PBS-treated** | **PGN-treated** |  | **Naive** | **PBS-treated** | **PGN-treated** |
| **stage** | **Parents** | **Parents** | **Parents** |  | **Parents** | **Parents** | **Parents** |
| **Larvae** |  |  |  |  |  |  |  |
| L2 | 2.7 ± 0.2 | 2.9 ± 0.1 | 2.2 ± 0.1 |  | 38 ± 1 | 47 ± 2 | 46 ± 1 |
| L3 | 3.4 ± 0.2 | 2.9 ± 0.1 | 2.9 ± 0.1 |  | 209 ± 6 | 240 ± 4 | 235 ± 6 |
| L4 | 4.2 ± 0.1 | 4.2 ± 0.1 | 4.3 ± 0.2 |  | 1511 ± 28 | 1544 ± 34 | 1527 ± 42 |
| L5 | 4.7 ± 0.1 | 5.0 ± 0.1 | 5.3 ± 0.1 |  | *11.0 ± 0.3* | *12.9 ± 0.4* | *12.6 ± 0.3* |
| Wandering larvae | 6.3 ± 0.1 | 6.8 ± 0.1 | 5.4 ± 0.1 |  | - | - | - |
| **Pupae** | 25.4 ± 0.4 | 26.2 ± 0.4 | 26.3 ± 0.1 |  | *6.0 ± 0.2* | *6.9 ± 0.1* | *6.7 ± 0.1* |
| **Adults** | 11.8 ± 0.9 | 12.7 ± 0.4 | 13.7 ± 0.6 |  | *2.4 ± 0.1* | *2.9 ± 0.1* | *2.9 ± 0.1* |

^1^ Weight: L2, L3, L4 weights specified in mg, *italic* numbers indicate L5, pupae and adults weights specified in g.

*N* = 18 for each developmental stage except for adults *N* = 9.

**Table S10.** Unchallenged offspring generation. Results of multiple post hoc *U*-tests with Benjamini-Hochberg correction which analysed the differences in the weight of *Manduca sexta* offspring between the different developmental stages (Fig. 4A, Table 4, significant offspring stage factor)

|  | L2 | L3 | L4 | L5 | 1P | A |
| --- | --- | --- | --- | --- | --- | --- |
| L2 | - |  |  |  |  |  |
| L3 | n.s. | - |  |  |  |  |
| L4 | *** | *** | - |  |  |  |
| L5 | n.s. | n.s. | *** | - |  |  |
| 1P | n.s. | n.s. | *** | n.s. | - |  |
| A | n.s. | n.s. | ** | n.s. | n.s. | - |

Offspring developmental stages: L2 – 2^nd^ instar, L3 – 3^rd^ instar, L4 – 4^th^ instar, L5 – 5^th^ instar, 1P – 1-day-old pupae, A – 1-day-old adults. Statistics: n.s. – not significant *P* > 0.05, ** *P* ≤ 0.01, *** *P* ≤ 0.001.

**Table S11.** Unchallenged offspring generation. Results of multiple post hoc *U*-tests with Benjamini-Hochberg correction which analysed the differences in developmental times of *Manduca sexta* between the different offspring stages and different parental treatments (naive, PBS, PGN) (Fig. 4B, Table 4, significant parental treatment x offspring stage factor)

|  |  | L2 | L3 | L4 | L5 | W | 1P | A | L2 | L3 | L4 | L5 | W | 1P | A | L2 | L3 | L4 | L5 | W | 1P | A |
| --- | --- | --- | --- | --- | --- | --- | --- | --- | --- | --- | --- | --- | --- | --- | --- | --- | --- | --- | --- | --- | --- | --- |
|  |  | N | N | N | N | N | N | N | PBS | PBS | PBS | PBS | PBS | PBS | PBS | PGN | PGN | PGN | PGN | PGN | PGN | PGN |
| L2 | N | - |  |  |  |  |  |  |  |  |  |  |  |  |  |  |  |  |  |  |  |  |
| L3 | N | n.s. | - |  |  |  |  |  |  |  |  |  |  |  |  |  |  |  |  |  |  |  |
| L4 | N | n.s. | n.s. | - |  |  |  |  |  |  |  |  |  |  |  |  |  |  |  |  |  |  |
| L5 | N | ** | n.s. | n.s. | - |  |  |  |  |  |  |  |  |  |  |  |  |  |  |  |  |  |
| W | N | n.s. | n.s. | ** | n.s. | - |  |  |  |  |  |  |  |  |  |  |  |  |  |  |  |  |
| 1P | N | * | n.s. | n.s. | n.s. | n.s. | - |  |  |  |  |  |  |  |  |  |  |  |  |  |  |  |
| A | N | n.s. | n.s. | n.s. | n.s. | n.s. | n.s. | - |  |  |  |  |  |  |  |  |  |  |  |  |  |  |
| L2 | PBS | n.s. | n.s. | n.s. | n.s. | n.s. | n.s. | n.s. | - |  |  |  |  |  |  |  |  |  |  |  |  |  |
| L3 | PBS | n.s. | * | *** | n.s. | *** | *** | n.s. | *** | - |  |  |  |  |  |  |  |  |  |  |  |  |
| L4 | PBS | n.s. | n.s. | n.s. | n.s. | ** | n.s. | n.s. | n.s. | *** | - |  |  |  |  |  |  |  |  |  |  |  |
| L5 | PBS | *** | n.s. | * | n.s. | * | *** | n.s. | *** | *** | * | - |  |  |  |  |  |  |  |  |  |  |
| W | PBS | ** | n.s. | n.s. | ** | * | ** | n.s. | n.s. | *** | n.s. | n.s. | - |  |  |  |  |  |  |  |  |  |
| 1P | PBS | * | n.s. | n.s. | n.s. | n.s. | n.s. | n.s. | n.s. | *** | n.s. | * | * | - |  |  |  |  |  |  |  |  |
| A | PBS | ** | n.s. | n.s. | n.s. | n.s. | n.s. | n.s. | * | *** | n.s. | n.s. | n.s. | n.s. | - |  |  |  |  |  |  |  |
| L2 | PGN | * | *** | ** | *** | *** | *** | ** | *** | * | ** | *** | *** | *** | *** | - |  |  |  |  |  |  |
| L3 | PGN | n.s. | * | *** | n.s. | *** | *** | n.s. | *** | n.s. | *** | *** | *** | *** | *** | * | - |  |  |  |  |  |
| L4 | PGN | n.s. | n.s. | n.s. | n.s. | ** | n.s. | n.s. | n.s. | *** | n.s. | n.s. | n.s. | n.s. | n.s. | ** | *** | - |  |  |  |  |
| L5 | PGN | *** | n.s. | ** | ** | ** | *** | n.s. | *** | *** | ** | n.s. | n.s. | ** | n.s. | *** | *** | n.s. | - |  |  |  |
| W | PGN | n.s. | ** | *** | *** | *** | *** | n.s. | *** | n.s. | *** | *** | *** | *** | *** | * | n.s. | *** | *** | - |  |  |
| 1P | PGN | *** | n.s. | * | n.s. | * | n.s. | n.s. | *** | *** | * | *** | n.s. | n.s. | n.s. | *** | *** | n.s. | *** | *** | - |  |
| A | PGN | *** | n.s. | * | ** | * | * | n.s. | *** | *** | * | n.s. | n.s. | * | n.s. | *** | *** | n.s. | n.s. | *** | * | - |

Offspring developmental stages: L2 – 2^nd^ instar, L3 – 3^rd^ instar, L4 – 4^th^ instar, L5 – 5^th^ instar, W – wandering stage, 1P – 1-day-old pupae, A – 1-day-old adults. Parental priming treatment: N – Naive untreated parents, PBS – control injection with PBS, PGN – immune priming with peptidoglycan injection, Statistics: n.s. – not significant *P* > 0.05, * *P* ≤ 0.05, ** *P* < 0.01, *** *P* < 0.001. Shaded area: Same developmental stages in the offspring of differently treated parents
